# Effects of Dietary Supplemental Chlorogenic Acid and Baicalin on the Growth Performance and Immunity of Broilers Challenged with Lipopolysaccharide

**DOI:** 10.3390/life13081645

**Published:** 2023-07-28

**Authors:** Huiyuan Lv, Peng Li, Zhiming Wang, Mingkun Gao, Guang Li, Wei Nie, Lei Xiao, Zengpeng Lv, Yuming Guo

**Affiliations:** 1 State Key Laboratory of Animal Nutrition and Feeding, College of Animal Science & Technology, China Agricultural University, Haidian District, Beijing 100193, China; lvhuiyuan0507@163.com (H.L.); mingkungao@outlook.com (M.G.); 18328415428@163.com (G.L.); caunw@163.com (W.N.); lvzengpeng310@cau.edu.cn (Z.L.); 2 Hubei Key Laboratory of Animal Nutrition and Feed Science, Wuhan Polytechnic University, Wuhan 430023, China; pengli@whpu.edu.cn; 3 Beijing Centre Biology Co., Ltd., Daxing District, Beijing 102218, China; 15313787686@163.com; 4 Hubei Lan Good Microbial Technology Co., Ltd., Yichang 443100, China; xiaol@lgzwbio.com

**Keywords:** chlorogenic acid, baicalin, broiler, immunity, growth performance

## Abstract

The objective of this study was to investigate the effects of dietary supplemental chlorogenic acid and baicalin (CAB) on the growth performance and immunity of broilers challenged with lipopolysaccharide (LPS). This study was designed as a factorial arrangement of 2 dietary CAB treatments × 2 LPS treatments. Birds challenged with or without LPS were fed with a basic diet (CON) and (LPS), the level of CAB diet containing 500 mg/kg CAB(CAB) and (CAB + LPS). The feeding trial lasted for 42 days. Results showed that there was a negative effect on average daily weight gain (ADG) and average body weight of broilers during the animal trial with LPS challenge. The levels of diamine oxidase (DAO), lysozyme (LYZ), immunoglobulin G (IgG), and IgA in the serum, the contents of IL-1β and TNF-α in the spleen were elevated with LPS treated. Additionally, LPS treatment tended to reduce the jejunal villi height (VH) and total superoxide dismutase (T-SOD) in the serum. Dietary supplemental 500 mg/kg CAB increased the body weight and ADG and improved the feed conversion ratio (FCR) during the trial period. In addition, dietary 500 mg/kg CAB elevated the ratio of VH to crypt depth in the jejunum and reduced the content of protein carbonyl. Beyond that, the levels of IgG and IgA in the serum and transforming growth factor (TGF-β) in the spleen were up-regulated with 500 mg/kg CAB supplementation. In conclusion, dietary CAB was beneficial for growth performance and immunity of broilers challenged with lipopolysaccharide.

## 1. Introduction

In the post-antibiotic era, some pathogenic microorganisms wait for an opportunity to proliferate. This poses a huge challenge for the farming industry. Pathogenic microorganisms in poultry production, such as *Escherichia coli*, *Clostridium perfringens*, *Salmonella*, and coccidium can elicit inflammatory responses in birds [1,2]. Inflammation can cause tissue damage, disrupt immunity, and enhance catabolism, which is a process that depletes nutrients [3,4]. The structural components of pathogenic microorganisms and the bacteriocins produced by their metabolism offer the opportunity for inflammation. For instance, lipopolysaccharide (LPS) is a component of the cell wall of some Gram-negative bacterial species, and it induces an inflammatory response in the body by activating the TLR4(Toll-like receptor 4)-NF-κB pathway [5,6]. Hence, it is urgent to develop new and stable feed additives to improve the health and growth performance of poultry, and some natural plant active ingredients are receiving a lot of attention here.

Honeysuckle and scutellaria are widely known as Chinese herbal medicines. They are mainly used to eliminate inflammation and relieve oxidative damage. However, it was not previously known what the main components were responsible for these effects. A study suggested that chlorogenic acid and baicalin are the biologically active ingredients in honeysuckle and scutellaria, respectively. Some in vitro studies demonstrated that chlorogenic acid helped alleviate the LPS-induced inflammatory response in macrophages [7,8]. Chlorogenic acid reduced the damage to vascular endothelial cells caused by acute increases in the Nitric oxide(NO) levels in intestinal mucosal microvascular endothelial cells that were challenged with LPS [9]. Baicalin is involved in regulating the metabolism of nitric oxide synthase, arachidonic acid, prostaglandin-2, and leukotrienes, thus contributing to resolving the inflammatory response [10]. Chlorogenic acid and baicalin are not only anti-inflammatory but also helpful to the growth performance of poultry. Studies suggested that either chlorogenic acid or baicalin has the potential for enhancing the growth performance and health of broilers [11,12]. Although the respective biological effects of chlorogenic acid and baicalin have been characterized, there are few reports on the combined efficacy of chlorogenic acid and baicalin.

We suspected that the combination of chlorogenic acid and baicalin might have an exciting effect on the growth performance and immune function of broilers. An inflammatory damage model is crucial for studying this content. LPS administration can establish such a model. Hence, the present study was designed to investigate the effects of dietary supplementation with the combination of chlorogenic acid and baicalin (CAB) on the growth performance and immunity of broilers challenged with LPS.

## 2. Materials and Methods

### 2.1. Experimental Design and Animal Management

All the procedures used in the present study were approved by the Animal Care Committee of China Agricultural University, Beijing, China (AW01703202-1-5).

**Figure 1:**
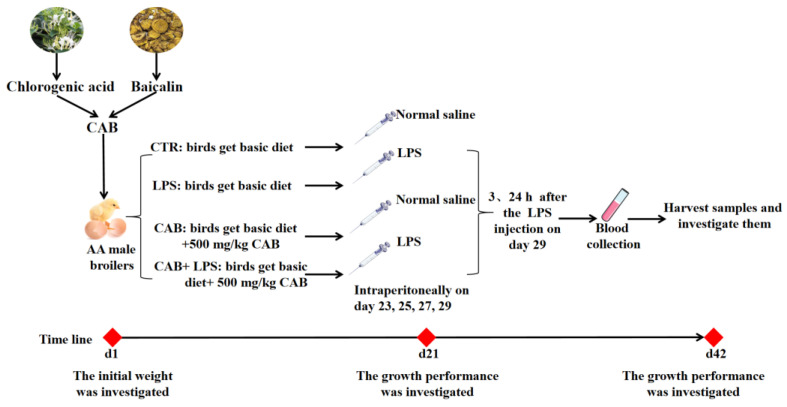
Flow chart of animal trial. The combination of chlorogenic acid and baicalin were used to treat the diet of broilers, and the LPS or normal saline were used to challenge the birds on day 23, 25, 27, and 29 of the animal trial. Afterward, 3 and 24 h after the LPS injection on day 29, the blood and samples were harvested.

The animal experiment was carried out at the Animal Experiment Base (Hebei) of China Agricultural University, China. The study was designed as a factorial arrangement of 2 dietary CAB treatments × 2 LPS treatments. A total of 320 1-day-old healthy AA male broilers with uniform weight (42.2 ± 0.8 g) were randomly assigned to four treatment groups, and each group consisted of 8 replicates and 10 birds each. The birds were treated with or without LPS, and their basic diet was supplemented with 0 or 500 mg/kg CAB. The basic diet was formulated according to the standard of NRC (2012), and the formula is shown in Table 1. LPS derived from *Escherichia coli* serotype O55:B5 was purchased from Sigma (Sigma‒Aldrich, St. Louis, MO, USA). Additionally, CAB was provided by Beijing Centre Biology Co., Ltd. (Beijing, China). The actual contents of chlorogenic acid and baicalin in CAB were 2.4 mg/g and 23.6 mg/g, respectively. The animal experiment lasted for 42 days, and broilers were weighed and performance was assessed on days 21 and 42 of the experiment. All the broilers were raised on-line, with free access to water and feed. The lighting conditions were 24 h of light for the first three days, 20 h of light and 4 h of darkness from days 4 to 7, and 18 h of light and 6 h of darkness, thereafter. The temperature control conditions were 35 °C for the first three days, followed by 33 °C for days 4 to 7, and then, the temperature was decreased by 1 °C every day until the temperature was maintained at 25 °C. Newcastle disease and infectious bronchitis vaccines were administered on day 8 of the experiment, and the bursal virus vaccine was administered on day 21. On days 23, 25, 27, and 29 of the animal experiment, the broilers in the LPS-treated group were intraperitoneally injected with LPS (1 mg/kg body weight, BW) [1], and the birds without LPS treatment received an equal volume of normal saline. 3 and 24 h after the last LPS injection, 1 bird in each replicate was selected, and blood was collected from the wing vein. Then, those birds were anesthetized via intravenous injection with 50 mg/kg BW sodium pentobarbital for slaughter sampling. Then, some indicators about intestinal barrier function, antioxidant capacity, and immune function were investigated. The animal trial was arranged according to Figure 1.

### 2.2. Growth Performance and Organ Index

The chickens were weighed after 8 h of fasting at 21 and 42 days of age, and the feed consumption was measured. Then, the average daily gain (ADG), average daily feed intake (ADFI), and feed conversion ratio (FCR) were calculated. Additionally, the thymus, spleen, and bursa of fabricius were weighed 24 h after the last LPS injection at 29 days of age, and the weight index of each organ was determined. Statistical analysis was performed according to the following formula: organ index = organ weight, g/living body weight, kg.

### 2.3. Jejunum Morphology

With the broiler anesthetized, the abdominal cavity was opened and the jejunum was taken out and the midpoint was found. Approximately 1 cm of the middle jejunum was collected from the chickens 24 h after the last LPS injection at 29 days of age. After fixation in 4% paraformaldehyde for 24 h, the jejunal sections were soaked through a graded series of ethanol and xylene and embedded in paraffin. The jejunum was then sectioned at 5 mm with a Lecia RM2235 microtome (Leica Biosystems Inc., Buffalo Grove, IL, USA). The sections were deparaffinized with xylene and rehydrated through a graded dilution of ethanol. Hematoxylin and eosin (H&E) staining. The jejunal villus height (VH) and crypt depth (CD) were measured according to the method described by Li [13], and the ratio of VH to CD was also determined. Briefly, eight intact intestinal villi with a straight direction were selected from each sample, and then the VH and CD were investigated. Villus height was defined as the distance from the villus tip to the crypt mouth, and crypt depth was the depth of the distance from the crypt mouth to the base.

### 2.4. Serum Levels of Diamine Oxidase, Immunoglobulins, and Antioxidant-Related Enzymes

Blood was harvested from the underwing vein 3 h and 24 h after the last LPS injection at 29 days of age. Then, the blood was centrifuged at 3000 r/min for 15 min at 4 °C, and the serum was separated and stored at −80 °C for later use. Kits from Nanjing Jiancheng Biotechnology Co., Ltd. (Nanjing, China) were used to measure the levels of diamine oxidase (DAO), lysozyme (LYZ), total superoxide dismutase (T-SOD), malondialdehyde (MDA), and protein carbonyl. Chicken ELISA kits purchased from Nanjing Jiancheng Biotechnology Co., Ltd. were used to measure the levels of immunoglobulin G (IgG), IgA, and IgM.

### 2.5. The Levels of Cytokines in the Spleen

With the broiler anesthetized, the abdominal cavity was opened and the spleens were harvested 3 h and 24 h after the last LPS injection at 29 days of age. Afterward, the spleens were promptly frozen in liquid nitrogen and stored at −80 °C for future utilization. A total of 0.1 g spleen and 0.9 mL prechilled saline were thoroughly mixed well in a 1.5 mL clean tube on ice. Then, the mixture was centrifuged at 3500 r/min for 15 min at 4 °C, and the supernatant was collected and stored at −80 °C for later use. Chicken ELISA kits purchased from Beijing Kangjia Hongyuan Biotechnology Co., Ltd. (Beijing, China) were used to investigate the levels of IL-1β, IL-2, IL-10, TGF-β, TNF-α, IFN-γ, IL-6, and IL-17.

### 2.6. Data Analysis

SPSS 23.0 software (SPSS Inc., Chicago, IL, USA) was used to analyze the data. Two-way ANOVA was performed in a 2 × 2 factor arrangement to analyze the effects of LPS treatment and CAB supplementation, as well as the interaction of these two factors. Then, a one-way ANOVA and Duncan’s multiple comparisons were performed to assess the interactions between LPS and CAB. *p* < 0.05 was considered a significant difference between different treatment groups.

## 3. Results

### 3.1. Growth Performance

**Figure 2:**
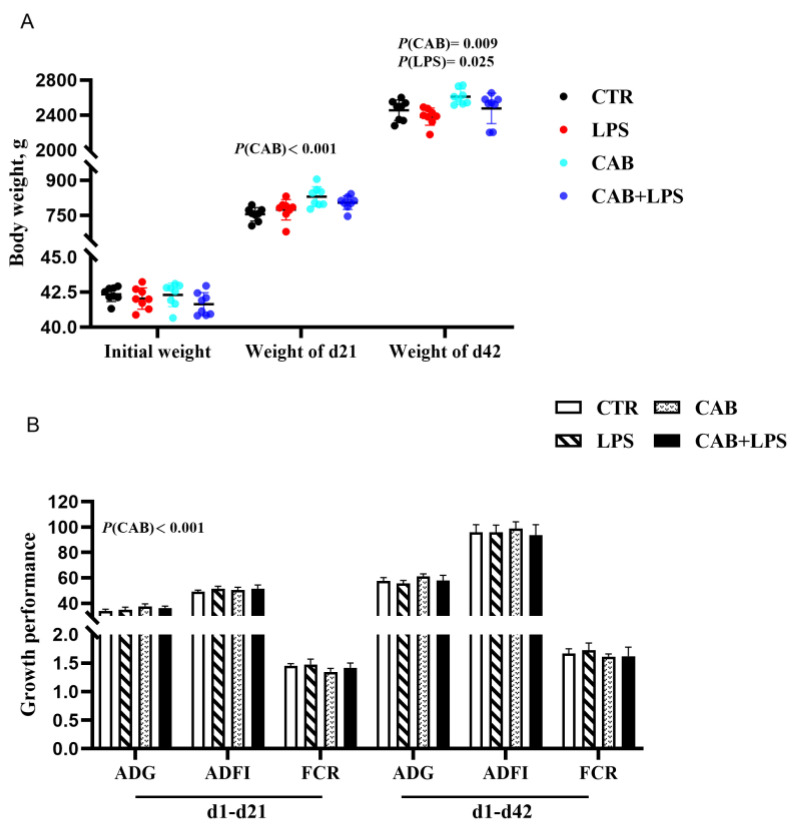
Effects of CAB on the growth performance of the birds challenged with LPS. The result of body weight was arranged in (**A**), and the results of ADG, ADFI, and FCR were shown in (**B**). The *p*-value of the main effect was indicated separately on each indicator.

The ADG from day 1 to day 21 of the animal trial was not affected by the LPS challenge, however, the ADFI and FCR during this time period tended to be negatively affected. In addition, the ADG during the entire trial period and the body weight (BW) at 42 days of age were decreased after the LPS challenge (*p* < 0.05) (Figure 2A,B). Dietary supplemental 500 mg/kg CAB improved growth performance from day 1 to day 21 and during the whole trial period. Specifically, the ADG, FCR from day 1 to day 21, and BW at 21 days of age were improved after treatment with CAB (*p* < 0.05) (Figure 2B). Additionally, the ADG and FCR throughout the trial period and the BW at 42 days of age also tended to be improved with 500 mg/kg CAB supplementation.

### 3.2. Intestinal Morphology and Organ Index

**Figure 3:**
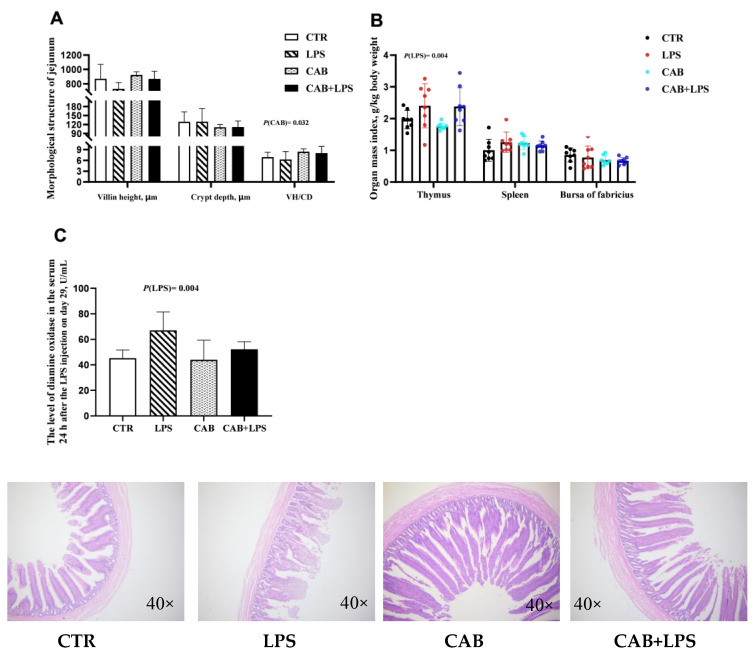
Effects of CAB on the intestinal morphological structure, immune organ mass index, and level of serum DAO of the birds challenged with LPS. The result of the intestinal morphological structure was arranged in (**A**), and the results of the immune organ mass index and the level of serum DAO were shown in (**B**,**C**), respectively. The *p*-value of the main effect was indicated separately on each indicator.

In the present study, we observed that LPS treatment decreased the jejunal willi height (*p* < 0.05) (Figure 3A), and CAB treatment increased the VH/CD ratio (*p* < 0.05). LPS treatment elevated the level of DAO (Figure 3C) in the serum. In addition, the mass index of the thymus was decreased with LPS challenge (*p* < 0.05) (Figure 3B). Dietary supplementation with 500 mg/kg CAB tended to decrease the level of serum DAO (*p* < 0.05) (Figure 3C).

### 3.3. Antioxidant and Immunity Function

**Figure 4:**
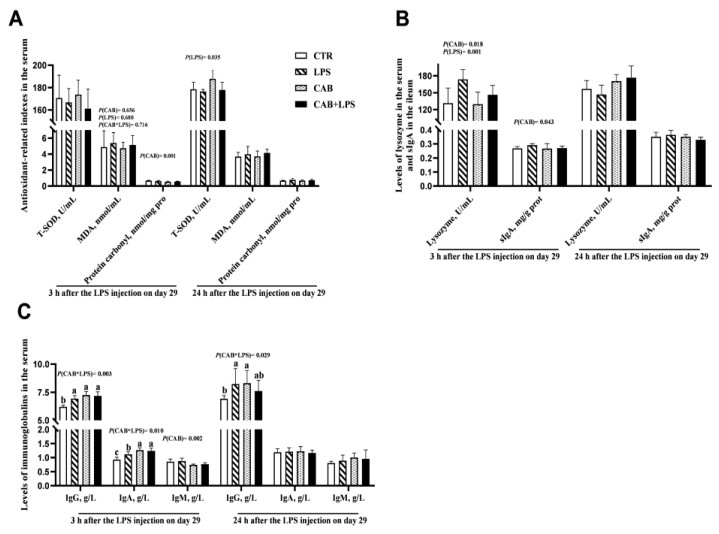
Effects of CAB on the antioxidant-related parameters and immune molecules of the birds challenged with LPS. The result of the antioxidant-related parameters was arranged in (**A**), and the results of serum lysozyme and ileal secretory immunoglobulin A (sIgA) were shown in (**B**). (**C**) shows the levels of serum Igs. The *p*-value of the main effect was indicated separately on each indicator. In each indicator, the columns marked with different letters indicate significant differences (*p* < 0.05). When an interaction effect occurs and the differences are significant, label them as a, b, c. When only the main effects are observed, label the *p*-value.

No significant effects of LPS on the activity of T-SOD and the level of MDA in the serum of broilers were observed 3 h after the last injection of LPS, and it was the same with CAB. The level of T-SOD in the serum was decreased 24 h after the last injection of LPS (*p* < 0.05) (Figure 4A). Interestingly, dietary supplementation with 500 mg/kg CAB helped to raise the level of T-SOD 24 h after the last injection of LPS and down-regulate the content of protein carbonyl 3 h after the last injection of LPS (*p* < 0.05). Additionally, the levels of lysozyme, IgG, and IgA 3 h after the last injection of LPS were increased (*p* < 0.05) (Figure 4B,C). Dietary supplementation with 500 mg/kg CAB increased the level of serum IgG, IgA, and IgM 3 h after the last injection of LPS (*p* < 0.05). It should be pointed out that those changes in these indices induced by LPS were not observed for 24 h after the last injection of LPS.

### 3.4. Spleen Cytokines mRNA Expression

**Figure 5:**
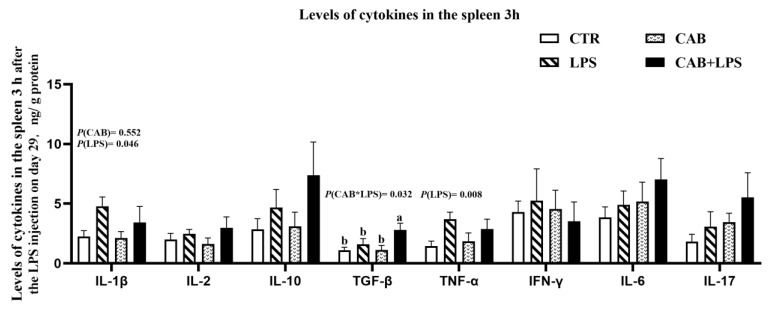
Effects of CAB on the cytokines in the spleen of the birds challenged with LPS (1). The results about the levels of cytokines in the spleen 3 h were arranged in this figure. The *p*-value of the main effect was indicated separately on each indicator. In each indicator, the columns marked with different letters indicate significant differences (*p* < 0.05).

**Figure 6:**
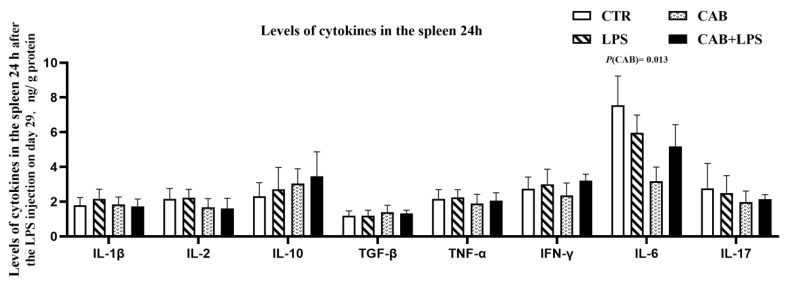
Effects of CAB on the cytokines in the spleen of the birds challenged with LPS (2). The results about the levels of cytokines in the spleen 24 h were arranged in this figure. The *p*-value of the main effect was indicated separately on each indicator (*p* < 0.05).

The spleen is an important immune organ in broilers, and we thought it would be appropriate to assess immune-related parameters in the spleen. In this study, we observed that the levels of some pro-inflammatory cytokines, such as IL-1β and TNF-α were upregulated in the spleen 3 h after the last injection of LPS (*p* < 0.05) (Figure 5). It was worth mentioning that dietary supplemental 500 mg/kg CAB contributed to up-regulating the level of TGF-β in the spleen (*p* < 0.05). The pro-inflammatory effect of LPS decreased with time after injection, as we observed that the levels of IL-1β and TNF-α were not changed by LPS for 24 h after the last injection of it (Figure 6). Dietary supplementation with 500 mg/kg CAB was able to down-regulate the level of IL-6. The regulatory effect of CAB on the immunity of broilers was manifested by upregulated expression of anti-inflammatory factors.

## 4. Discussion

A large number of studies have suggested that the intraperitoneal injection of LPS is a reliable method for establishing a model of inflammation in broilers [14,15]. Some inflammatory cytokines are secreted to activate the body’s immune response to LPS treatment, and inflammatory response is a process of consuming nutrients [14]. Additionally, LPS facilitates the abnormal transcription of appetite-related genes, such as proopiomelanocortin (POMC) and cocaine- and amphetamine-related transcript peptide (CART), which in turn exert negative effects on feeding [16]. In the present study, the growth performance of broilers was negatively affected by LPS challenge. This suggested that the LPS-induced model was successfully established. A multitude of plant extracts have been used to improve the growth performance and health of broilers as an alternative to antibiotics; however, no single plant extract is currently in use for this purpose. Hence, a strategy that combines several substances would be more potential. A study suggested that chlorogenic acid has the potential to improve the growth performance and antioxidant function of broilers [12]. The effect of baicalin on the growth performance of broilers has rarely been reported, but it can improve broiler health [17]. In this study, we observed that the combined use of chlorogenic acid and baicalin contributed to the growth performance of broilers. In addition, it seemed that this improvement was more pronounced in the early stage of growth. The intestinal barrier function and immune function of broilers were in the mature stage at that time, and they were also easily disrupted by environmental factors, and the effect of CAB on growth performance might be attributed to its ability to mitigate this disruption. Growth performance is regulated by many factors, which not only involves the category of nutrition but also needs to understand the beneficial effects of CAB on the growth performance of broilers from other perspectives such as physiology and immunity [18,19]. The following study was designed to investigate it accordingly.

The intestine is not only the main place for the digestion and absorption of nutrients but also the largest immune organ of the body. An intact intestinal structure is the basis of immune defense, and high levels of DAO in the serum might indicate that the intestinal barrier has been disrupted [20]. In our study, the level of DAO was elevated after the LPS challenge, and there was also the negative effect of LPS on the intestinal morphological structure. Interestingly, dietary supplementation with CAB decreased the level of DAO in the serum. The ideal intestinal structure is the material basis of intestinal barrier function, and a huge number of studies demonstrated that higher intestinal villi are more helpful for growth performance [21,22]. In the present study, the jejunal villi height was increased after CAB supplementation with 500 mg/kg, and this might contribute to the better growth performance of broilers. Although the indicators that we evaluated were limited, the available evidence suggested that CAB might improve the growth performance of broilers by enhancing gut health, and the mechanism needs to be further studied.

The oxidative stress is a manifestation of the imbalance between the body’s oxidation and antioxidant systems. Inflammation, aging, and abnormal environmental stimulation are the accomplices of oxidative stress. Once oxidative stress occurs in the body, some peroxide products such as malondialdehyde, protein carbonyl, and hydrogen peroxide are produced in large quantities, thus causing tissue damage. Some antioxidant enzymes such as superoxide dismutase and catalase are expressed to alleviate it. Some studies have suggested that chlorogenic acid and baicalin are useful for elevating the levels of superoxide dismutase, glutathione peroxidase, and catalase, and down-regulating the contents of malondialdehyde and myeloperoxidase, which contributed to improving the antioxidant capacity in the body [23,24]. In the present study, the level of protein carbonyl was decreased after supplementation with 500 mg/kg CAB. The natural structural characteristics of chlorogenic acid and baicalin could reduce the content of oxygen-free radicals and superoxide anions. This might be one of the reasons for the improved antioxidant function of broilers. This outcome is consistent with previous studies [25]. The effect of CAB on antioxidant function in broilers might be another evidence of its improved growth performance.

A multitude of natural plant active ingredients play the role of regulating the body’s immune function, which is the main reason why it contributes to alleviating the inflammatory response. As we mentioned above, suppressing the inflammatory response prevents the depletion of nutrients, thus ensuring the conversion and deposition of nutrients in the poultry [26]. This is also strong evidence that those natural plant active ingredients improve poultry growth levels. Hence, the effect of CAB on immune function in broilers was investigated. The main immune organs of poultry include the thymus, spleen, and bursae of fabricius. They are sensitive sites stimulated by corresponding inflammatory factors. In the present study, the mass index of the thymus was increased after LPS treatment, which was consistent with what others have reported [27]. This result demonstrated that the immune system of broilers was activated by LPS challenge. Lysozyme in serum is considered to be a factor that is involved in the innate immune system [28], and immunoglobulins also play a key role in protecting the body from stress [29]. In this study, the levels of lysozyme and IgG in the serum were elevated after LPS treatment. lysozyme and IgG are secreted by immune cells in response to LPS challenge, dietary supplementation with 500 mg/kg CAB increased the levels of IgG, IgA, and IgM in the serum. Our finding likely revealed that dietary CAB was able to enhance the immunity of broilers. To investigate it further, a follow-up study was conducted.

Anti-inflammatory cytokines, such as IL-10, TGF-β, and IL-4, and proinflammatory cytokines, such as IL-1β, IL-6, and TNF-α, are involved in maintaining the immune homeostasis of the body [30]. The LPS-mediated inflammatory response induces the secretion of proinflammatory cytokines [31], and anti-inflammatory cytokines are produced in response to inflammatory responses. In the present study, the levels of IL-1β, TNF-α, and TGF-β in the spleen were elevated after LPS challenge. There was no doubt that LPS elicited a vigorous immune response in the spleen, and enlargement of the spleen was inevitable in this study. It should be noted that dietary supplementation with 500 mg/kg CAB increased the level of TGF-β, and decreased the content of IL-6 in the spleen of birds challenged with LPS. TGF-β is mainly secreted by regulatory T cells and Th2-type cells, and some pro-inflammatory cytokines, such as IL-1β, IFN-γ, and IL-6 are mainly secreted by Th1-type cells. Based on our outcomes, it could be concluded that CAB might contribute to the differentiation of Th cells into Th2-type and Treg cells. With the Th2-type and Treg cells proliferated and differentiated, the anti-inflammatory cytokines were secreted to regulate immune homeostasis. Which helped alleviate the pro-inflammatory response induced by LPS treatment. As stated above, dietary supplementation with CAB raised the levels of immunoglobulins, we hold that CAB might enhance the cross-talk of Th2-type cells and B lymphocytes. This was very meaningful in the inflammatory response induced by LPS. Further studies are needed to reveal the underlying mechanisms here.

Although a detailed investigation of the effect of CAB on growth performance and health is beyond the scope of this work, we acknowledged that CAB has the potential to improve it. Hence, it deserves further study. Given the widespread interest in the industry, it is also expected that new, stable, and efficient feed additive products targeting poultry health and production levels will be developed in the future based on the combination of chlorogenic acid and baicalin.

## 5. Conclusions

The utilization of a dietary combination of chlorogenic acid and baicalin exhibited promising effects on improving the growth performance, antioxidant capacity, and immunity of broilers under inflammatory conditions. This finding not only highlights the potential for using plant extracts as feed additives but also provides novel research perspectives and directions in this field.

## Figures and Tables

**Table 1:** Test diet composition and nutrition level, % (air-dry basis).

Items	d 1–d 21	d 22–d 42
Corn, 7.8% protein	56.73	59.95
Soybean meal, 46% protein	31.80	27.40
corn gluten meal, 44% protein	2.50	2.50
Cottonseed meal, 43.5% protein	2.00	2.50
Soybean oil	2.88	4.00
CaHPO₄	1.81	1.51
Stone powder	1.28	1.22
NaCl	0.35	0.35
DL-Methionine, 98%	0.21	0.12
L-Lysine HCl, 78%	0.12	0.13
Vitamin premix ①	0.02	0.02
Mineral premix ②	0.20	0.20
Choline chloride, 50%	0.10	0.10
Total	100	100
Nutrition levels ③		
ME (MJ/kg)	12.34	12.76
Crude protein, %	21.50	19.50
Calcium, %	1.00	0.90
Total phosphorus, %	0.71	0.64
Available phosphorus, %	0.45	0.40
Lysine, %	1.10	1.00
Methionine, %	0.50	0.40
Methionine+ Cystine, %	0.92	0.76

① Vitamin premix (provided per kilogram of feed) the following substances: vitamin A, 12,500 IU; vitamin D3, 2500 IU; vitamin E, 18.75 mg; vitamin K3, 2.65 mg; vitamin B1, 2 mg; vitamin B2, 6 mg; vitamin B12, 0.025 mg; biotin, 0.0325 mg; folic acid, 1.25 mg; nicotinic acid, 50 mg; pantothenic acid, 12 mg. ② Trace element premix (provided per kilogram of feed) the following substances: Cu, 8 mg; Zn, 75 mg; Fe, 80 mg; Mn, 100 mg; selenium, 0.15 mg; iodine, 0.35 mg. ③ Calculated value based on the analyzed data of the test diet.

## Data Availability

The data of this study were presented in the article, and no additional sequencing data here. If necessary, please consult the corresponding author politely.
